# Editorial: Advanced technological applications in neurosurgery

**DOI:** 10.3389/fsurg.2023.1277997

**Published:** 2023-09-06

**Authors:** Chao-Hung Kuo, Tsung-Hsi Tu, Ko-Ting Chen

**Affiliations:** ^1^ Department of Neurosurgery, Neurological Institute, Taipei Veterans General Hospital, Taipei, Taiwan; ^2^ School of Medicine, National Yang Ming Chiao Tung University, Taipei, Taiwan; ^3^ Department of Biomedical Engineering, National Yang Ming Chiao Tung University, Taipei, Taiwan; ^4^ School of Medicine, Chang Gung University, Taoyuan, Taiwan; ^5^ Department of Neurosurgery, Chang Gung Memorial Hospital, Taoyuan, Taiwan; ^6^ Neuroscience Research Center, Chang Gung Memorial Hospital, Taoyuan, Taiwan

**Keywords:** exoscope, focus ultrasound, BCI = brain-computer interface, EDAS = encephaloduroarteriosynangiosis, navigation, neurosciece, neuro-signaling

**Editorial on the Research Topic** Advanced technological applications in neurosurgery

The development of new technology changes several ways in our daily life, and improves the precision and quality of medicine, as well as in the field of neurosurgery. The application of robotic system in neurosurgery is one of example, which has been shown to improve surgical precision, particularly in complex procedures, such as intracranial biopsies and the placement of spinal instrumentation and intracranial leads (1). Not only improved the clinical outcomes, the advance to technology could cease the obstacle of residents training. Exoscopes have also been developed to assist surgeons by identifying anatomical structures that lie outside the surgical field (Calloni et al.). The goal of this research topic would focus on different technological applications on from brain to spine, and understanding how the impacts on the filed of neurosurgery.

Recent advances in methodologies can be seen in collateral circulation (extracranial and intracranial), which has been shown to improve the prognosis of patients undergoing the neurosurgical procedures. Surgical revascularization plays an key role in the treatment of ischemic cerebrovascular disease (2), and recent indirect methods are proving simpler and less risky than direct anastomosis in terms of bypass vessels occlusion and overperfusion (3, 4). One recent example of indirect revascularization is the encephalo-duro-arterio-synangiosis method developed by Nuerlanbieke et al. for patients with hemodynamic disorders resistant to conventional medical treatment. The establishment of extracranial and extracranial collateral circulation was observed circulation in the operation area and improve the prognosis of patients.

The enhancement of instrumentation improves the surgical outcomes. For the intracranial lesion, focused ultrasound (FUS) could modulate transiently the permeability of the blood-brain barrier. The bioavailability of therapeutic agents is site-specifically augmented only in the zone where the FUS energy is targeted (5). The targeted and reversible blood-brain barrier opening break the mold of therapeutic applications in managing central nervous system disorders (6). Researchers have also combined a frameless FUS system with advanced neuronavigation systems for the treatment of patients with recurrent glioblastoma (7). On the other hand, surgical microscopes have greatly enhanced the precision of spinal surgery (8) leading to notable improvements in clinical outcomes (9, 10). To improve the localization during surgery, Diepers et al. designed the patient-mounted Cube Navigation System, which could improve accuracy of guiding needle placement for complex access routes in lumbar pain therapy. The Cube Navigation System has the potential to improve needle guidance for complex access routes, especially considering the ease of use of the device. The CT-guided navigation system was not only used on the cranial or spinal surgery, but the CT-guided thoracic sympathetic nerve block and radiofrequency was also proved an effective treatment for the patients with the primary palmar hyperhidrosis (Zhang et al.).

Further advanced technology focused not only improvement of clinical outcome, but recovery neurological deficits via rehabilitation with training by the exoskeleton (11, 12). Nonetheless, there is a gap in our knowledge of biophysiological functions and their effects on functional performance (13, 14). The analysis of neuro-signals help us realize a non-linear relationship between the cortical signal and the motor output for some, but importantly not all, movement types (13). In the other study, the role of motor inhibition was observed in both inferior frontal gyri, with age-related lateralization to the right side, which was proved in the previous functional magnetic resonance imaging studies. The evidence correlation of age and response inhibition was observed directly by the evidence of cortical recordings (14). The quantitative measurements of cortical signals would improve our understandings the relationship between neural outputs and physiological behaviors. The analysis provides insight into the tuning of motor cortex toward specific types of motor behaviors, and further application on the brain-computer interface.

Cancer neuroscience is an emerging research field which represents the relationship between the nervous system and cancer, from the initiation, progression, and therapeutic-induced reaction (15). Not only primary malignant brain tumors, such as gliomas, but also brain metastases have been linked to the glutamatergic-contributed tumor growth (16–18). The impact of glutamatergic neurotransmitter activity on tumor behavior is only part of the story, the complex interaction between neurons and brain tumor cells has been inferred as “neuron-to brain tumor synaptic communication” (19). The crosstalk between neuron-glioma cells and the organization of glioma functional networks are now being recognized as highly relevant for tumor growth, invasiveness, and resilience to chemo-radiotherapies (16, 17, 20, 21). To think a step further, potential strategies for therapeutic disconnection of the resilience networks have been proposed (19, 22). Other potential targets including inhibition of malignant synaptogenesis, exploitation of synaptic connectivity, or inhibition of neuronal hyper-excitability, are being actively studied (22). To this end, the complex tumor microenvironment is becoming more sophisticate yet with more therapeutic potential since new mechanisms of tumor resistance are discovered.

The advances in science and technology have driven the development of precision medicine, bringing medicine to a new level in diagnosis and treatment. In neurosurgery, more studies including interdisciplinary collaborations in biomedical engineering, which combine the strengths of medicine and engineering would lead the development of neuroscience and neurosurgery to a new era.
